# Determinants of adherence to the Mediterranean diet among individuals with type 2 diabetes mellitus living in Mediterranean countries: a systematic review

**DOI:** 10.3389/fnut.2025.1523995

**Published:** 2025-02-03

**Authors:** Janot J. Ayoub, Suzan A. Haidar, Ellen E. Blaak, Nanne K. De Vries

**Affiliations:** ^1^NUTRIM School of Nutrition and Translational Research in Metabolism, Department of Health Promotion, Maastricht University, Maastricht, Netherlands; ^2^Department of Nutrition, Faculty of Nutrition and Health Sciences, Lebanese International University (LIU), Beirut, Lebanon; ^3^Department of Human Biology, NUTRIM Institute of Nutrition and Translational Research in Metabolism, Maastricht University, Maastricht, Netherlands; ^4^Department of Health Promotion, CAPHRI and NUTRIM, Faculty of Health, Medicine, and Life Sciences, Maastricht University, Maastricht, Netherlands

**Keywords:** sociodemographic, type 2 diabetes, Mediterranean diet, health promotion, nutrition therapy, self-efficacy

## Abstract

**Background:**

Patients with type 2 diabetes mellitus (T2DM) are often encouraged to adopt a healthy diet, such as the Mediterranean Diet (MD) yet limited evidence exists about adherence. An increased shift toward a “Western” dietary pattern was observed.

**Objective:**

This systematic review aims to gain insight into the various factors that may enhance or reduce adherence to the MD in patients with T2DM residing in Mediterranean countries.

**Method:**

We retrieved published studies from 2000 to 2023 from PubMed, Cochrane, Embase, CINAHL, Web of Science, Medline, and PsycINFO databases. The criteria for inclusion included individuals residing in Mediterranean countries aged 18+ with T2DM who underwent assessment using a validated MD scoring tool. We included 17 studies, with participant numbers ranging from 106 to 7,447.

**Results:**

Compliance with the MD was moderate, with the most significant determinants of adherence being age, physical activity, body mass index (BMI), marital status, and educational level. However, limited information is available on psychological and economic determinants.

**Conclusion:**

Various factors, especially sociodemographic ones, influence adherence to the MD among patients with T2DM. Future research should explore economic and psychological factors that may significantly impact adherence.

**Systematic review register:**

Prospero: CRD42023396094.

## Introduction

The Mediterranean Diet (MD), initially described in the 1960s, is a dietary pattern rich in vegetable oils and low in saturated fats, primarily associated with Greece and Southern Italy (1). It is a diet rich in monounsaturated fat, fiber, antioxidants, vitamins, and minerals (2–4). The diet encourages a relatively high intake of fat, up to 40% of total energy, primarily from olive oil, along with whole unprocessed grains, fruits, vegetables, pulses, and nuts; a moderate to high consumption of fish; a moderate to low consumption of white meat and skimmed dairy products; a low amount of red meat and meat products; and a moderate consumption of wine with meals (5, 6).While various definitions of the MD exist, most share these core principles, agreeing on nutrient content rather than on food quantities, highlighting a distinct advantage to defining the diet by nutrients rather than food (2).

Populations in the olive-growing regions of the Mediterranean basin have long consumed MD (7). Due to its plant-based origin, positive impact on the ecosystem, as well as its economic and sociocultural dimensions, it was acknowledged as a “sustainable diet” in 2010 (8). In his famous “Seven Countries Study,” Ancel Keys documented the health benefits of the MD in the prevention of cardiovascular diseases (CVD) (9). Many other researchers, have analyzed the associations between dietary habits and chronic diseases, with the majority of results favoring beneficial MD dietary patterns (10–12). Non-Mediterranean countries have since advocated for the use of MD.

Approximately 90–95% of all diagnosed cases of diabetes are T2DM, a progressive disease that often manifests long before diagnosis (13). The current worldwide prevalence of diabetes mellitus is approximately 425 million people (14). The increased prevalence of T2DM has led to its recognition as a devastating progressive disease that accelerates aging and causes both microvascular and macrovascular complications (15).

Studies have shown that MD is suitable for the overall management of T2DM, as it is associated with better glycemic control than other diets (16, 17). It possesses anti-inflammatory and antioxidant properties and affects human energy and substrate metabolism (18). MD can impact blood glucose levels by reducing insulin resistance, thus enhancing diabetes management (19–21). The PREDIMED study revealed that an MD rich in olive oil reduces the incidence of CVD compared with a low-fat diet in high-risk patients, including those with diabetes (22). Esposito et al. (23) also concluded that the MD is beneficial for the lipid profile and helps control glycated hemoglobin A1c (HbA1c) levels in people with T2DM. Furthermore, adherence to MD dietary patterns has been associated with better quality of life and increased life expectancy for individuals with T2DM (24).

Unfortunately, the literature reveals that those living in Mediterranean countries no longer adhere to the MD (25). In a systematic review performed by Obeid et al. (26), adherence to an MD in Mediterranean countries was found to be low to moderate.

Numerous factors influence withdrawal from an MD, with economic factors being at the top of the list (27). Psychosocial, environmental, and demographic factors also influence adherence to an MD (28–30).

Since the rates of T2DM are on the rise and adherence to an MD is declining and given the beneficial effect of the diet in managing diabetes and preventing associated complications, this systematic review aims to gain a better understanding of factors that can either increase or decrease adherence to MD in patients with T2DM who live in Mediterranean countries. The information obtained will be crucial in developing interventions and nutrition policies to improve compliance.

## Methodology

In accordance with the PRISMA guidelines (31) (Preferred Reporting Items for Systematic Review and Meta-analysis), a systematic review of papers reporting on determinants of adherence to the MD among patients with T2DM living in Mediterranean countries was carried out, and the review protocol was registered in the PROSPERO database under the registration number CRD42023396094.

### Selection criteria for studies

Studies including adults (aged over 18 years) with T2DM who resided in a Mediterranean country (i.e., Albania, Algeria, Bosnia, Croatia, Cyprus, Egypt, France, Gibraltar, Greece, Israel, Italy, Lebanon, Libya, Morocco, Malta, Monaco, Montenegro, Palestinian territory, Slovenia, Spain, Syria, Turkey, and Tunisia) and used a validated dietary assessment and scoring tool to measure adherence to the MD were eligible for inclusion. The selection included clinical trials and observational studies (cohort, case–control, and cross-sectional) published between November 2000 and February 2023, as the MD began to gain attention and was linked to chronic diseases in early 2000 (32). Only articles published in English were reviewed. Reviews, qualitative articles, conference abstracts, commentaries, unpublished studies, letters to editors, dissertations and posters were excluded. Studies that assessed adherence among participants with diseases such as renal failure, inflammatory bowel disease, hepatic disease, lung disease, wasting disease Human Immunodeficiency Virus (HIV) or cancer, and gestational diabetes were also excluded. The outcomes of interest included behavioral, psychological, demographic, and personal factors affecting adherence to the MD. We also reported disparities in adherence between genders and groups of varying socioeconomic statuses.

### Literature search

A sensitive and specific search strategy was created with the help of a medical librarian. The search strategy included three key concepts: (1) adherence, (2) a Mediterranean diet, and (3) type 2 diabetes mellitus. For each concept, medical subject headings (MeSH) and keywords were mapped according to the instructions offered in each electronic database. Some of the terms included in the search were compliance, barrier, determinants, dropout, obstacles, satisfaction, attitudes, adaptation, MEDAS, MD, Med-diet, T2DM, noninsulin-dependent diabetes mellitus (NIDDM), and hyperglycemia. The search strategy was run in the following databases: MEDLINE-PubMed- Embase-CINAHL- PsycINFO-Cochrane Library-Web of Science. Appendix S2 contains all the search strategies and searches. The most recent publication date was February 2023. In September 2024, the search was conducted again on PubMed, yielding no new articles that fit the inclusion criteria.

**Figure 1:**
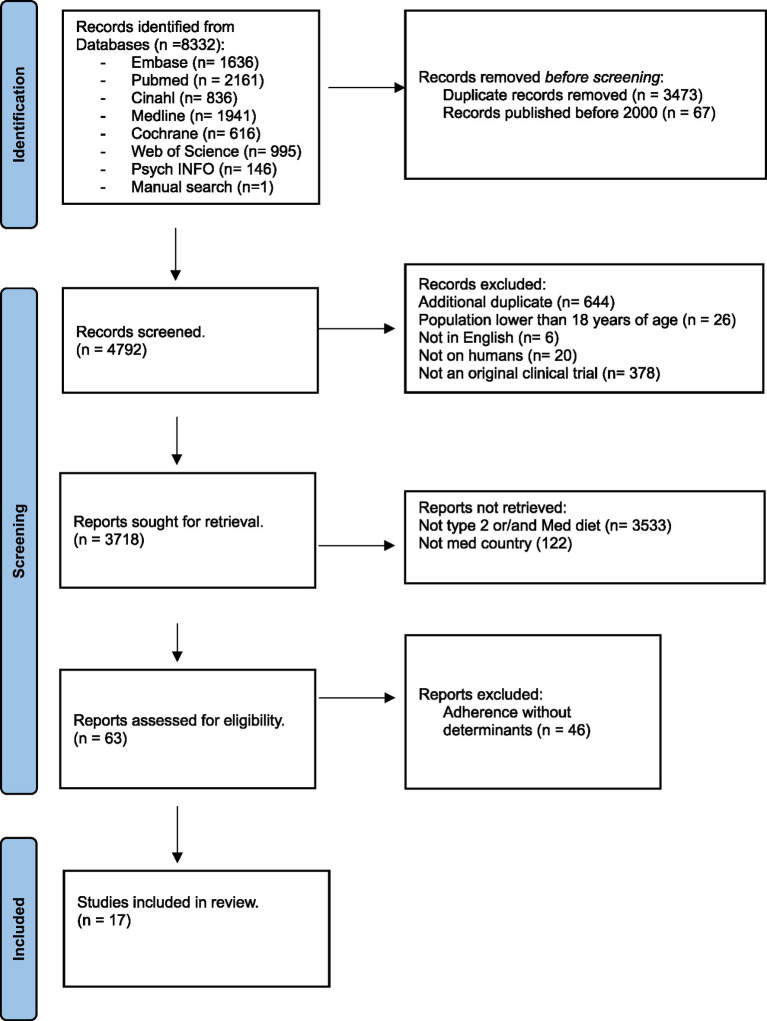
PRISMA diagram of study selection.

We extracted all records into EndNote software, version 20.5. Articles published before 2000 and those published in duplicate were discarded by JA. Screening was performed by two independent reviewers, (JA) and (SH), using the predetermined inclusion and exclusion criteria. The kappa score was calculated to be 0.63, indicating substantial agreement. In cases of discrepancy, a third reviewer (N.D.V./E.B.) was invited to resolve the conflict. After reaching an agreement, the reviewers independently identified the relevant articles and eliminated additional duplicates that Endnote software had not previously detected. The two reviewers retrieved all potentially eligible articles and conducted a full-text screening. The final number of articles was 17 (Figure 1). To obtain more information on certain studies, some authors, such as Martinez and El Achhab, were contacted via email.

### Data extraction and quality assessment

Two authors (JA and SH) independently extracted data from the full-text articles. They recorded the authors, the year of publication, the sample size, the study settings, and the study design. They also recorded information about the participants, such as their age, sex, marital status, economic status, level of education, nationality, BMI, and smoking status. Furthermore, they documented the instruments used to assess adherence to the MD, including their respective cutoff points and the mean adherence to the diet (Table 1). Finally, they reported and classified the determinants of adherence to the MD as negatively related, positively related, or not associated with MD adherence. The reviewers discussed any discrepancies until they reached 100% agreement. The Appendix S1 contains a detailed data extraction table.

**Table 1:** Mean adherence scores with the MD scoring tool and its respective classification in the included studies.

References–Country	MD soring tool	Range of MD adherence score for classification in categories	Mean adherence (Mean score ± SD)
Martinez-Gonzalez et al. (22)–Spain	MEDAS	Low (score ≤ 5). Moderate (score 6–9). High (score ≥ 10)	8.6 ± 2 for the general population, 8.5 ± 2 for those with T2DM, 8.5 ± 2 for females, and 8.7 ± 2 for males.
El Achhab et al. (28)–Morocco	MEDAS	Low (score 0–7). High (score 8–14)	7.8 ± 1.2. 59% of the sample had a score of ≥8 points.
Vidal-Peracho et al. (38)–Spain	MEDAS	Low (score ≤ 5). Moderate (score 6–9). High (score ≥ 10)	8.7 ± 1.82 for the whole population. 8.5 ± 1.81 for those with T2DM.
Roldan et al. (5)–Spain	MEDAS	Low ≤8. Good ≥9	6.2 ± 0.62 preeducation. 6.8 ± 0.62 post education. A score of 6.5 for women and 5.8 for men
Kudret et al. (24)–Turkey	MEDAS	Low (score ≤ 5). Moderate (score 6–9). High (score ≥ 10)	No mean adherence score. 39.0% of patients had low compliance. 56.5% had moderate compliance, and 4.5% had high compliance. 30% of men had low compliance versus 46.4% of women, 65.6% of men had moderate compliance versus 56.5% of women, and 4.4% of men had high adherence versus 4.5% of women.
Yilmaz and Yangilar (40)–Turkey	MEDAS	Low (score ≤ 5). Moderate (score 6–9). High (score ≥ 10)	The median PREDIMED score was 6. 46.3% had low adherence, 51.7% had medium and 2.0% had high.
Sanchez-Hernandez et al. (42)–Spain	MEDAS	Low-to-moderate (score < 9 points) or moderate-to-high (Score ≥ 9 points).	The mean score of MEDAS was not reported. 49.4% have poor/moderate adherence. 49.3% of men versus 49.4% of women had poor/moderate adherence.
Downer et al. (39)–Spain	MEDAS	High (score ≥ 11) and low (score < 11)	At 1-year follow-up, the mean score for low adherence was 8.2 ± 1.8 and the mean score for high adherence was 9.3 ± 1.8. 82% of patients had low compliance at 2-year follow up and 81% had low adherence at 3-year follow-up. At the 4-year follow-up, the mean score for low adherence was 8.5 ± 2 and the mean score for high adherence was 9.3 ± 9.
Badrasawi et al. (35)–Palestine	MEDAS	Low adherence (score ≤ 5); moderate adherence (score between 6 and 9); and high adherence (score ≥ 10).	53.8% had low/moderate categories of adherence and 46.2% had high adherence categories. The average score of the high adherence group was 10.8 ± 0.9, while that of the low/moderate group was 7.4 ± 1.4. 59% of the male patients reported high adherence compared to just 38.8% of the females.
Muñoz-Pareja et al. (37)–Spain	MEDAS	score from 0 to 14 with moderate accordance given to a score of ≥7.	MEDAS mean score was 6.8 (95% CI 6.65–7.02), and 57.4% (95% CI 52.6–62.3%) had accordance with MEDAS. Excluding alcohol, the MEDAS score increased to 74.9% (95% CI 70.3–79.3%).
Giugliano et al. (36)–Italy	9-point scale adapted by Trichopoulo et al. (7)	3 subgroups: low (score of 0–3), middle (4, 5), and high (6–9).	No mean adherence to the Med Diet was reported. A total 28% had low adherence, 48% had moderate adherence and 24% had high adherence.
Bonaccio et al. (41)–Italy	9-point scale adapted by Trichopoulo et al. (7)	3 subgroups: low (score of 0–3), middle (4, 5), and high (6–9).	Overall mean adherence was not mentioned. A total 30% of the population had poor adherence, 44% had moderate adherence and 26% had high adherence. The percentage of men was 66.1% of whom 27% had low adherence, 45.3% moderate, and 27.8% high.
Ortega et al. (44)–Spain	Panagiotakos score (ATTICA study)	A score of 5 to 0 for each item. Low adherence (score < 23); Medium (between 23 and 26); high (>26).	The mean (±SD) med score was 24 ± 5. Of the general population, 36.5% had a low adherence (57% were women); 31% had a medium adherence (60% were women) and 32.4% had a high adherence (54% female)
Alcubierre et al. (45)–Spain	Alternate Mediterranean Diet (aMED) score.	aMED: includes nine key components of the traditional MD: The score ranges from 0 (minimal adherence) to 9 (maximal adherence)0.3 tertiles: T1 (score of 0–3); T2 (score of 3–6) and T3 (score of 6–9)	The mean aMED score was: 4.3 (1.5). Of 238 patients with type 2 Diabetes: 30.3% were in T1; 47.9% in T2 and 21.8% in T3.
Grahovac et al. (29)–Croatia	Med Diet service score (MDSS)	A score from 0 to 24. A score of over 13.5 points is considered good adherence to MD principles.	The median score of adherence is 8.0 (6.0–10.0). (9.9%) patients fulfilled the criteria for adherence to MD.
Bucan Nenadic et al. (39)–Croatia	Mediterranean Diet Serving Score (MDSS).	Score from 0 to 24. Optimal adherence with a score > 14pts	The mean MDSS score was 8.3 ± 3.45. 8.9% scored 14 or more points on total MDSS. 8.3% of patients with T2DM versus 9.1% of patients with CKD and T2DM had a score of 14 or more on MDSS.
Petroni et al. (30)–Italy	Med diet score (MDS-11 items)	Optimal, good, fair, scarce, and low at the respective cut-offs: ≥45; 44–34; 33–23; 22–12; ≤ 11	MDS score was 29.6 ± 5.7 in the total population. 29.6 ± 5.4 in women and 29.7 ± 6 in men.

Quality assessment was conducted using the Newcastle–Ottawa scale (NOS) adapted for cross-sectional (33) studies and another version for cohort studies (34). The NOS assesses three main domains related to the selection procedure (sample size and appropriateness, non-responder characteristics and ascertainment of exposure), comparability of subjects in different outcome groups, and outcome assessment. The cross-sectional assessment assigned a maximum of 5 points for selection, 2 points for comparability, and a maximum of 3 points for the outcome, resulting in a total grade of 10. We applied the same criteria to the cohort studies, except for 4 points for the first domain, which led to a total score of 9 points. We used the following scoring algorithm to rate the quality of the studies: a total score of 8–9 points (10 for cross-sectional studies) indicated a low risk of bias, a score of 7 or 6 points indicated a medium risk of bias, and a score of 5 points or less indicated a high risk of bias. Table 2 displays the results of the quality assessment. JA and SH independently performed the quality assessment. We discussed disagreements until we reached a consensus.

**Table 2:** Quality assessment of the included studies based on the Newcastle Ottawa scale (NOS).

Studies (cross-sectional and observational)	Selection (maximum five stars)	Comparability: (maximum two stars)	Main outcome (adherence to med diet) (maximum three stars)	Final score/10	Risk of bias
El Achhab et al. (28)	2	1	2	5	High
Grahovac et al. (29)	5	2	2	9	Low
Giugliano et al. (35)	3	2	2	7	Medium
Vidal-Peracho et al. (36)	4	2	2	8	Low
Roldan et al. (5)	2	2	2	6	Medium
Kudret et al. (24)	2	2	2	6	Medium
Yilmaz and Yangilar (37)	3	1	2	6	Medium
Sanchez-Hernandez et al. (38)	4	1	2	7	Medium
Bucan Nenadic et al. (39)	4	2	2	8	Low
Ortega et al. (40)	5	2	2	9	Low
Badrasawi et al. (41)	5	1	2	8	Low
Alcubierre et al. (42)	4	2	2	8	Low
Muñoz-Pareja et al. (43)	3	1	2	6	Medium
Martinez-Gonzalez et al. (22)	5	2	2	9	Low
Downer et al. (44)	4	2	2	8	Low

Studies (Cohort)	Selection	Comparability: (Maximum 2 stars)	Outcome	Final score/9	Risk of bias
Bonaccio et al. (45)	3	2	3 (4 years)	8	Low
Petroni et al. (30)	2	2	3 (2 years)	7	Medium

### Data analysis

We grouped predictors of adherence into three categories for this review: demographic and personal variables, general health and behavioral determinants, and socioeconomic and physical determinants (Table 3). The following sections detail the determinants based on these categories. Note that the included articles did not assess determinants related to the patient’s psychological status, such as readiness, avoidance, indifference, and self-blame.

**Table 3:** Categories of determinants affecting MD adherence in T2DM.

Demographic and personal determinants	General health and behavioral determinants	Socioeconomic and physical determinants
Gender	History of associated diseases	Habitation
Age	Physical activity (PA)	
Education	BMI	
Marital status	Waist circumference (WC)	
Therapeutic education	Duration of Diabetes	
	Smoking	
	Alcohol	
	Chronotype	
	Total energy intake	

## Results

### Characteristics of the included studies

The search of the seven databases yielded 17 studies that met the selection criteria. Among the 17 studies, eight were conducted in Spain (5, 22, 36–44), two of which (22, 44) performed a cross-sectional analysis on the same population (PREDIMED sample) but with different outcomes. Three studies were conducted in Italy (30, 35, 45), two other studies were conducted in Turkey (24, 37), two were conducted in Croatia (29, 39), one study was performed in Morocco (28)^,^ and one was conducted in Palestine (41). The number of participants in the included articles was between 106 and 7,447. The ages ranged from 18 to 88 years. The percentage of males included in the studies varied between 24 and 66%, with 100% in one of the studies (35) that assessed erectile dysfunction in males with T2DM. Two studies Petroni et al. (30) and Bonaccio et al. (45) were cohort studies, whereas the remaining 15 were cross-sectional.

In some studies, researchers reported the mean adherence to the MD score or the percentage of adherence for both the general population and each category of determinants. Researchers have used several scoring tools to assess MD adherence. Although each of these scores used a different adherence range to the MD, the scale interpretation was positive for healthy items and negative for unhealthy items, with high scores indicating better adherence than the lowest scores. Table 1 provides an overview of the MD scoring tools and their respective cutoff points.

### Quality analysis of the included studies

According to the NOS, the quality assessment of the studies included in this analysis yielded the following results: among the 15 cross-sectional studies reviewed, one study El Achhab et al. (28) exhibited a high risk of bias due to incomplete selection criteria; six studies were deemed to have a moderate risk for insufficient sample characteristics and confounding variable adjustment; and eight had low risk and were primarily from Spain.

For the cohort studies, one had low risk, whereas the other had moderate risk, both lacking details on the non-exposed cohort and one missing outcome specification at the start of the study (Table 2).

### MD adherence in patients with type 2 diabetes

To assess adherence to the MD, 10 studies employed the MEDAS tool. The average adherence scores across these studies ranged from 6 to 8.54, indicating low to moderate adherence, with Turkey reporting the lowest mean (37) and Spain reporting the highest (36). Notably, one of these studies carried out in Palestine (41) opted to substitute alcohol in the scoring tool with water intake. This substitution acknowledges the religious constraints that prohibit alcohol consumption, emphasizing the importance of respecting such cultural considerations. Besides MEDAS, other MD scoring tools were also used and are detailed in Table 1.

Studies performed in Spain revealed stable adherence levels from 2012 to 2017, with MEDAS scores ranging from 8.2–8.77. However, in 2019, adherence decreased to 6.22 (5), followed by a slight improvement in 2020 (38, 42). Notably, in Spain, adherence to the MD in the general population experienced a significant decline between 1987 and 1997, coinciding with the rise of the Western diet; however, adherence began to stabilize in 1998, with a slight recovery after that (46). Similarly, Italy exhibited a significant decline in MD adherence from 1985 to 1986 and 2005–2006, with better adherence in southern Italy (47). In the present review, a slight improvement in adherence was observed between 2010 and 2016 in two studies performed in southern Italy (35, 45). In Croatia, less than optimal adherence was observed in the included studies, a fact often realized in many Croatian studies that showed a shift from the MD in recent decades, with moderate adherence associated with increased overweight rates (48, 49). Notably, during the economic crisis of 2007–2008, adherence to the MD decreased further in Croatians, highlighting the importance of financial status as a strong determinant of healthy eating patterns (50). In Turkey, moderate adherence to the MD was observed among T2DM patients, mirroring trends in the general population (51). Conversely, Morocco struggles with universal MD adoption, showing a loss of adherence in the general population (52), a pattern also evident among T2DM patients, as seen in the present review. Finally, in Palestine, moderate adherence to the MD was found among T2DM patients, reflecting broader Mediterranean trends (41).

### Determinants of adherence to the MD in patients with T2DM

#### Demographic and personal variables

Eleven papers examined the relationship between sex and adherence to an MD, seven of which reported no significant correlation (5, 24, 30, 36–42). However, two studies reported a positive correlation between male sex and adherence (45, 41). Muños Pareja et al. (43) reported a negative association between female sex and adherence, which vanished when they excluded wine consumption from the MEDAS score. In a Spanish study, a negative association with the female gender at baseline before the “education intervention” was found which reversed after a 3-year follow-up, highlighting the role of education in increasing adherence (44).

In terms of age, four studies out of nine reported a positive relationship between advanced age and adherence to the MD (28, 40–43).

Six studies examined the correlation between education level and adherence to an MD. Of these, four (22, 30, 38, 40) identified a significant positive association with higher education, whereas two studies (24, 44) did not find a significant link. Despite the absence of statistical significance, Kudret et al. (24) reported a better quality of life among university graduates with T2DM, which was often linked to improved adherence to the MD. Sánchez et al. (38) reported that highly educated patients, in addition to displaying increased MD adherence, exhibited a better compliance with oral antidiabetic agents used in diabetes treatment. Notably, the included studies categorized education into primary, secondary, and university levels, along with an additional category for illiteracy. Furthermore, a general observation across studies (22, 24, 38) indicated that males tend to have a higher education level than females.

Finally, marital status appeared to significantly influence adherence to the MD, with married individuals exhibiting greater adherence (22, 40).

#### General health and behavioral determinants

A positive association between PA and compliance with the MD was identified in all studies that investigated the association (22, 28, 38–42, 44–45). Various tools have been used to assess the frequency and intensity of PAs (Appendix S1). According to Downer et al. (44), PA remained a significant predictor of adherence to the MD at baseline and throughout the 4-year follow-up.

Six studies investigated the relationship between BMI and MD adherence. Four of these studies (5, 29, 40, 35) reported a negative association between high BMI and adherence, whereas two did not (24, 42). Researchers reported that a high BMI predicted poor adherence to an MD, and conversely, greater adherence to an MD resulted in weight reduction and a lower BMI (5, 29). BMI was calculated by dividing the weight in kilograms by the square of height in meters, and values were interpreted using standard categories for males and females defined by the WHO (53). Notably, a high BMI was consistently associated with a high waist circumference (WC), indicating a heightened level of abdominal obesity (29, 40, 35).

With respect to WC, studies have shown that patients with T2DM who exhibited high MD adherence had lower WC (40, 44, 35). Conversely, adherence to this diet contributed to a reduction in WC (40). Badrasawi et al. (41) discovered a link between abdominal obesity and reduced PA and physical function, ultimately resulting in a lower quality of life among patients with diabetes.

Five studies investigated the effect of diabetes duration as a determinant influencing MD adherence. Three of them indicated the absence of a significant correlation (5, 28, 36), whereas two others confirmed the presence of a relationship between diabetes duration and MD adherence (29, 45). Grahovac et al. (29, 45) reported that patients with a longer duration of T2DM adhered more closely to the MD guidelines than those who were recently diagnosed, whereas Bonaccio et al. (45) reported higher adherence in those who were recently diagnosed with diabetes.

#### Socioeconomic and physical determinants

Habitation or residence was identified as one of the determinants influencing adherence to the MD, as assessed by El Achhab et al. (28). In their study, they categorized habitation into rural, suburban, and modern areas. Interestingly, adherence to the MD was more significant in suburban areas than in rural and modern areas.

### Other determinants

Other determinants less frequently mentioned were related to a history of cardiovascular diseases, CVD (22, 44), total energy intake (22, 44), existing diseases (28, 39), diet education (5, 40), chronotype category (37), smoking (24, 40, 35) and alcohol (22, 24, 28, 41).

Researchers reported a positive correlation between diabetes and comorbidities, such as hypertension, and MD adherence (28) but reported no significant association between chronic kidney disease and MD adherence in individuals with T2DM (39). On the other hand, Downer et al. (44) and Martinez et al. (22), who analyzed PREDIMED data, reported that having more CVD risk factors predicted poorer adherence to the MD. Furthermore, they both concluded that low energy intake during short-and long-term follow-ups was associated with poor adherence to an MD.

With respect to alcohol intake, Martinez et al. (22) reported a positive association with MD adherence. However, Kudret et al. (24) did not find a significant correlation between alcohol intake and MD adherence. On the other hand, El Achhab et al. (28) reported that alcohol consumption limits adherence, particularly in “older patients with diabetes,” who typically receive advice from their caregivers to limit alcohol consumption (28). During their research in Palestine, Badrasawi et al. (41) did not include alcohol consumption in the MEDAS scoring tool. The Palestinian population’s cultural and religious context, which prohibits alcohol, led to this omission. Instead, they emphasized water intake as a substitute for alcohol in their assessment of MD adherence.

According to Giugliano et al. (35) and Kudret et al. (24), no significant association exists between smoking and MD adherence in individuals with T2DM. However, Ortega et al. (40) reported a significant negative correlation between smoking and MD adherence.

Roldan et al. (5). Observed a moderate increase in adherence to MD following educational intervention, justified by Ortega et al. (40), who also found that diet education may play a role in increasing adherence to MD.

Yilmaz and Yangilar (37) conducted a study to explore the possible connection between chronotype and MD adherence in a Turkish population, where 8.3% had T2DM. The study found no correlation between chronotype and MD adherence, as morning, intermediate, and evening types did not differ in their compliance with this diet (37). Morningness was defined as getting up early and starting with the daily activities; in the evening chronotype, people prefer to wake up late and start with the activities in the afternoon and evening, whereas intermediate type chronotype is the type between morning and evening chronotypes.

## Discussion

The findings of this review revealed positive correlations between advanced age, marital status, lower BMI, higher education, PA, suburban habitation and adherence to the MD. On the other hand, results suggest a negative correlation between smoking and adherence to the MD, as well as conflicting results regarding male sex, alcohol consumption, diabetes duration, chronotype, and MD adherence.

The positive correlation with male sex may be explained by the fact that females in the Mediterranean countries typically have numerous family responsibilities and commitments, leaving them with less time to focus on their health and adhere to healthy diets imposed by diabetes, including adherence to the MD, whereas males were found to be more adherent to dietary restrictions (41). Furthermore, the lack of a clear association between female sex and MD may, in part, be attributed to the use of scoring tools that do not account for the difference in alcohol consumption between males and females, as both genders are often given the same cutoff points, favoring males over females in assessment (42). Nevertheless, most of the studies reported in this paper indicate an absence of an association between sex and adherence to an MD in patients with T2DM. In a systematic review performed by Obeid et al. (26) to assess adherence to an MD in the general Mediterranean population, few differences were observed between sexes, and most of the included studies reported low to moderate adherence. This finding was also supported by Kyriacou et al. (54). To build upon these results, formulate and tailor adequate sex-specific interventions, further studies should be conducted in this field.

The association with age is likely due to cultural factors and the fact that older people tend to cook and eat more at home than younger persons with type 2 diabetes, who often stick to a more Westernized dietary pattern (40, 43). This finding was also supported by Veronese et al. (47), who assessed trends in adherence to an MD in southern Italy.

With respect to marital status, marriage increases adherence to an MD, which is well supported in the literature (55). In the review conducted by Goodridge et al. (55), it was demonstrated that married life offers a sanctuary of social and emotional connectedness between spouses, exerting a positive or healing effect on individuals with T2DM.

Education is positively related to adherence in most studies, which is a plausible association, as educated patients tend to read and seek more information on how to address their disease (22, 30, 38, 40). In a study conducted by Mogre et al. (56) to assess the factors associated with self-care behaviors in patients with T2DM, age, sex, and education were the most important variables impacting self-care. Nevertheless, some studies in this review did not relate these determinants to adherence, and an insignificant association was reported between these demographic variables and adherence (5, 24, 36, 38).

Concerning smoking, when a significant association was found, this variable was negatively linked to MD adherence. This may be explained by the observation that smokers typically do not follow a healthy lifestyle and may thus disregard adherence to a healthy diet (40).

Two studies in this review have raised concerns about alcohol consumption among patients with T2DM who adhere to an MD. These studies propose gender-specific guidelines for alcohol consumption, suggesting that females consume less alcohol than males (28, 41). Moreover, in many Mediterranean countries, alcohol consumption is forbidden due to religious beliefs. This highlights the necessity of identifying substitutes for alcohol in scoring mechanisms and modifying the scoring system accordingly.

In the category of general health and behavioral determinants, PA was positively correlated with MD adherence in all the studies that included this variable. This association can be partly attributed to a causal relationship, as patients with T2DM are typically advised to increase their PA for better glycemic control and self-care (28, 35). Increasing PA is also known to improve physical function and quality of life in patients with diabetes (28, 42).

With respect to BMI, patients with higher BMIs were found to be less adherent to the MD than those with a lower BMI (5, 29, 40, 35). Like patients with PA, patients with T2DM are often advised to reduce their weight, as this can help improve insulin sensitivity and subsequently the diabetes profile and associated complications, which may contribute to this positive relationship between lower BMI and increased adherence to an MD.

The relationship between diabetes duration and adherence to an MD has been controversial, with some studies reporting the existence of a significant association but others reporting that it does not exist. According to the literature, studies (57, 58) support the findings that strict dietary habits often fade with time, as shown in the article by Bonaccio et al. (59), who reported higher adherence in those who were recently diagnosed with diabetes than in those with a longer diagnosis duration. According to Austin et al. (57) and Ko et al. (58) patients with diabetes remain under threat of diabetes complications despite being adherent to self-care behaviors, which may result in burnout. Therefore, psychological and emotional support should be provided to these patients while providing them with constructive feedback, self-care guidance, and clear expectations of health outcomes to reinforce autonomy and self-efficacy (57).

In the socioeconomic category, suburban habitation was associated with better adherence to the MD. El Achhab et al. (28) assessed this determinant on a small sample of participants in a Moroccan study, limiting the generalizability of the results. Nevertheless, the NOS evaluates El Achab et al.’s study as low-quality, thereby diminishing the significance of its findings. However, a possible explanation for the relationship between MD adherence and urban habitation might be that those living in an urban area have frequent and easier access to health information via healthcare facilities and media, which will help them better understand their disease and treatment options (60, 61).

Although this review revealed no association between chronotype and MD adherence, many other studies that investigated this relationship found the opposite to be true. In a study assessing MD adherence in obese individuals, those with an evening chronotype were not only less adherent to the diet but also more likely to follow unhealthy dietary patterns, skip breakfast, engage in less physical activity, have higher BMIs, and be mostly smokers (62). Raiha et al. (63) conducted another study that strongly correlated the evening chronotype with low productivity and low income, potentially influencing food choices and adherence to healthy dietary patterns. Finally, researchers have reported that the chronotype significantly disrupts the circadian rhythm and increases the appetite for sugar and unhealthy items in patients with T2DM, leading to abnormal glycemic control (64, 65). These data suggest the need for further studies on the impact of chronotype on MD adherence in patients with T2DM.The studies reviewed did not assess the direct relationship between economic status and adherence to the MD. It is widely acknowledged that the cost of food plays a significant role in food purchasing decisions, as populations with lower incomes tend to prefer calorie-dense and more processed foods because of their affordability (49, 66, 67) and hence exposing themselves to a greater risk of diet-related diseases (66).

Unfortunately, the included studies did not assess cognitive or psychological determinants. Given the significant impact of self-efficacy on self-regulation techniques, which include goal setting, preparation for action, and subsequent behavior (68, 69), it could be beneficial to conduct additional high-quality research to identify potential barriers to MD adherence and promote self-efficacy in diabetes management, especially among those with long-term T2DM.

This review included only studies conducted in Spain, Italy, Croatia, Turkey, Morocco, and Palestine. However, an absence of studies concerning African Mediterranean countries (Egypt, Lybia, Tunisia, and Algeria) and Middle Eastern regions (Syria, Lebanon, and Israel) has been reported in the literature. This underscores the need for further research in these regions, which are known for their healthy traditional MD. Such research should consider factors like alcohol consumption and develop scoring tools tailored to the cultural, traditional, and economic contexts of these countries, as many of them face significant economic challenges that may impede adherence to MDs because of the relatively high cost of products associated with this dietary pattern (29).

### Strengths and limitations

This SR has several strengths. It followed the PRISMA guidelines (Appendix S3) and was registered in the Prospero database (CRD42023396094). Multiple databases were searched (MEDLINE-PubMed- Embase-CINAHL- PsycINFO-Cochrane Library-Web of Science) to identify all pertinent, peer-reviewed manuscripts. In addition, screening, data extraction, and quality assessment were performed independently by two authors, and the tools used to assess the quality of the included studies were validated (70).

However, our SR is not without limitations. Initially, the search was restricted to studies published in the English language. Additionally, a variety of scoring tools have been employed to assess MD adherence, each utilizing different classification systems. This discrepancy, as assessed by an epidemiologist, made it unfeasible to compare adherence across studies and aggregate the results for conducting a meta-analysis. Furthermore, conclusive results lack determinants concerning their impact on adherence, as many insignificant relationships have been reported. Additionally, socioeconomic and psychological factors were not assessed in the included studies. Finally, the included studies had a low to moderate risk of bias, particularly in terms of general population representativeness, non-respondent characteristics, and the MD adherence scoring tool. This highlights the need for higher-quality studies to gain a deeper understanding of MD adherence in Mediterranean communities with T2DM, which can help in tailoring appropriate interventions.

To our knowledge, this is the first systematic review that seeks to identify, evaluate, and summarize findings regarding adherence to an MD among adults with T2DM residing in Mediterranean countries. In summary our data indicate that a variety of critical factors, including age, sex, physical activity, education level, BMI, diabetes duration, waist circumference, and marital status, influence generally moderate adherence rates. Notably, we identified a significant gap in the literature with respect to the psychological and cognitive factors that influence MD adherence, which represents a critical area for future research. Moreover, we did not address economic factors as determinants of MD adherence in T2DM patients, which prompted new research in this field.

## Conclusion

Considering the results obtained from this systematic review, we recommend creating a “tailored MD scoring tool” specifically for patients with T2DM that would modify alcohol consumption guidelines to account for sex-specific and cultural factors. Additionally, it is essential to allocate resources to health promotion efforts to improve MD adherence among patients with T2DM. Therefore, it is imperative to facilitate referrals to dietetic professionals who can evaluate patient progress, encourage self-efficacy in meal planning, promote daily PA, and offer comprehensive support for adhering to the MD.

Together, these strategies have the potential to significantly enhance dietary adherence and improve the health of individuals with type 2 diabetes.

## Data Availability

The original contributions presented in the study are included in the article/Supplementary material, further inquiries can be directed to the corresponding author.
