# Supplementary material for: Determinants of adherence to the Mediterranean diet among individuals with type 2 diabetes mellitus living in Mediterranean countries: a systematic review
Source: Front Nutr. 2025 Feb 3;12:1523995. doi: 10.3389/fnut.2025.1523995 (PMC11830624; doi:10.3389/fnut.2025.1523995)
Supplement: Supplementary file 2 [file Table_2.docx]

**Appendix- S2**

**Search Strategy**

1. Database: CINAHL

| S1 | (MH "Nutritional Counseling") |
| --- | --- |
| S2 | (MH "Patient Education+") |
| S3 | TI (Patient N5 (satisfaction or education or behavio?r or attitude or preference or accept* or refusal)) OR AB (Patient N5 (satisfaction or education or behavio?r or attitude or preference or accept* or refusal)) |
| S4 | AB (dropout or drop-out or complian* or noncomplian* or non-complian* or adher* or nonadher* or non-adher*) OR TI (dropout or drop-out or complian* or noncomplian* or non-complian* or adher* or nonadher* or non-adher*) |
| S5 | (MM "Guideline Adherence") OR (MM "Treatment Failure") OR (MM "Treatment Withdrawal") |
| S6 | (MM "Health Beliefs") OR (MH "Patient Compliance+") OR (MH "Patient Satisfaction+") OR (MH "Attitude to Obesity") OR (MH "Adaptation, Psychological+") OR (MH "Eating Behavior+") OR (MH "Habits+") OR (MH "Social Behavior+") OR (MM "Attitude to Change") |
| S7 | (MM "Behavioral Changes") OR (MM "Consumer Attitudes") |
| S8 | (TI temperance or AB temperance) |
| S9 | AB (barrier* or obstacle* or difficult* or reason* or factor*) OR TI (barrier* or obstacle* or difficult* or reason* or factor*) |
| S10 | AB (enabler* or facilitator* or determinan* or motivator*) OR TI (enabler* or facilitator* or determinan* or motivator*) |
| S11 | S1 OR S2 OR S3 OR S4 OR S5 OR S6 OR S7 OR S8 OR S9 OR S10 |
| S12 | ((MH "Diet+" or MH "Diet, Reducing") AND (TI mediterran* or AB mediterran*)) |
| S13 | AB (diet* and mediterran*) OR TI (diet* and mediterran*) |
| S14 | TI (MD) or AB (MD) |
| S15 | TI (TEAM-MED or MEDAS or med-diet) OR AB (TEAM-MED or MEDAS or med-diet) |
| S16 | TI (mediterranean N5 (lifestyle or life-style)) OR AB (mediterranean N5 (lifestyle or life-style)) |
| S17 | S12 OR S13 OR S14 OR S15 OR S16 |
| S18 | \| TI (T2DM or niddm or diabet*) OR AB (T2DM or niddm or diabet*) \|  \| \| --- \| --- \| |
| S19 | MH "Diabetes Mellitus, Type 2") OR "Diabetes Mellitus, Type 2" OR (MH "Glucose Metabolism Disorders+") |
| S20 | (MH "Hyperglycemia+") |
| S21 | TI (hyperglycaemi* or hyperglycemi*) OR AB (hyperglycaemi* or hyperglycemi*) |
| S22 | S18 OR S19 OR S20 OR S21 |
| S23 | S11 AND S17 AND S22 |

1. Database: Cochrane
2. [mh "guideline adherence"] OR [mh "Treatment Adherence and Compliance"] OR [mh "adaptation, psychological"] OR [mh attitude] OR [mh behavior] OR [mh "consumer behavior"] OR [mh "health behavior"] OR [mh "personal satisfaction"] OR [mh ^"social behavior"] OR [mh "psychology, social"] OR [mh "psychosocial functioning"] OR [mh temperance] OR [mh "psychological phenomena"] OR [mh "behavioral disciplines and activities"]
3. (barrier* OR obstacle* OR difficult* OR enabler* OR facilitator* OR determinan* OR motivator*):ti,ab,kw
4. [mh counseling] OR [mh "behavior therapy"] OR [mh "patient education as topic"] OR [mh "patient compliance"] OR (Satisfaction OR education OR behavior* OR behaviour* OR attitude OR preference OR Dropout OR "drop-out" OR complian* OR noncomplian* OR "non complian*" OR adher* OR nonadher* OR "non adher*" OR accept* OR refusal OR reason* OR factor*):ti,ab,kw
5. #1 OR #2 OR #3
6. ("med-diet" OR MD OR "TEAM-MED" OR MEDAS OR mediterranean NEAR (lifestyle OR "life-style")):ti,ab,kw
7. (diet* AND mediterran*):ti,ab,kw
8. [mh "diet, mediterranean"]
9. [mh diet] AND mediterran*:ti,ab,kw
10. #5 OR #6 OR #7 OR #8
11. (T2DM OR niddm OR diabet*):ti,ab,kw OR [mh "Diabetes Mellitus"] OR [mh "diabetes mellitus, type 2"] OR [mh Hyperglycemia] OR (hyperglycaemi* OR hyperglycemi*):ti,ab,kw
12. # 4 AND #9 AND #10
13. Database: Embase

--------------------------------------------------------------------------------

1 exp nutritional counseling/ or patient counseling/ or behavior therapy/ or health education/ or exp diabetes education/ or exp nutrition education/

2 (Patient* adj5 (satisfaction or education or obstacle or motivator or counseling or knowledge or behavio?r* or attitude or preference)).tw.

3 (dropout or drop-out or complian* or noncomplian* or non-complian* or adher* or nonadher* or non-adher* or accept* or refusal).tw.

4 exp patient dropout/ or exp patient engagement/ or exp patient participation/ or exp patient preference/ or exp patient satisfaction/ or exp refusal to participate/

5 patient compliance/ or exp dietary compliance/ or psychological adjustment/ or adaptation/ or adaptive behavior/ or exp dietitian attitude/ or attitude to health/ or *health behavior/

6 exp consumer attitude/ or attitude to change/ or customer satisfaction/ or behavior change/ or health behavior/ or health belief/ or social preference/

7 temperance.tw.

8 or/1-7

9 exp Mediterranean diet/

10 (diet* and mediterran*).tw.

11 MD.tw. or (mediterranean.tw. and exp diet/)

12 (TEAM-MED or MEDAS or med-diet).tw.

13 (mediterranean adj5 (lifestyle or life-style)).tw.

14 or/9-13

15 (T2DM or niddm or diabet*).tw.

16 diabetes mellitus/ or exp diabetic obesity/ or exp impaired glucose tolerance/ or exp lipoatrophic diabetes mellitus/ or exp non insulin dependent diabetes mellitus/

17 exp hyperglycemia/

18 (hyperglycaemi* or hyperglycemi*).tw.

19 or/15-18

20 8 and 14 and 19

1. Database: Ovid MEDLINE(R)

1 exp Counseling/

2 exp Behavior Therapy/

3 exp Patient Education as Topic/

4 (satisfaction or education or behavio?r* or attitude or preference).mp.

5 (dropout or drop-out or complian* or noncomplian* or non-complian* or adher* or nonadher* or non-adher* or accept* or refusal or reason* or factor*).mp.

6 exp Guideline Adherence/ or exp "Treatment Adherence and Compliance"/ or exp Patient Compliance/

7 exp adaptation, psychological/ or exp attitude/ or exp behavior/ or exp consumer behavior/ or exp health behavior/ or exp personal satisfaction/ or social behavior/ or exp psychology, social/ or exp psychosocial functioning/ or exp temperance/ or exp psychological phenomena/ or exp "behavioral disciplines and activities"/

8 (barrier* or obstacle* or difficult*).mp.

9 (enabler* or facilitator* or determinan* or motivator*).mp.

10 exp Patient Compliance/

11 or/1-10

12 exp diet/ and mediterran*.mp.

13 exp Diet, Mediterranean/

14 (diet* and mediterran*).mp.

15 MD.tw.

16 (TEAM-MED or MEDAS or med-diet).mp.

17 (mediterranean adj5 (lifestyle or life-style)).mp.

18 or/12-17

19 (T2DM or niddm or diabet*).mp.

20 exp Diabetes Mellitus, Type 2/

21 exp Hyperglycemia/

22 (hyperglycaemi* or hyperglycemi*).mp.

23 or/19-22

24 11 and 18 and 23

1. Database: PsycInfo

| S1 | (counseling) OR (DE "Educational Counseling") |
| --- | --- |
| S2 | DE "Disease Management" OR DE "Health Literacy" OR DE "Healthy Eating" |
| S3 | (adapt* or satisfaction or education or behavio?r or attitude or preference or accept* or refusal) |
| S4 | (dropout or drop-out or complian* or noncomplian* or non-complian* or adher* or nonadher* or non-adher*) |
| S5 | DE "Dietary Restraint" |
| S6 | DE "Compliance" OR DE "Treatment Compliance" OR DE "Client Attitudes" OR DE "Treatment Barriers" OR DE "Treatment Refusal" |
| S7 | DE "Client Satisfaction" |
| S8 | DE "Environmental Adaptation" |
| S9 | DE "Behavior" OR DE " Refusal" OR DE "Healthy Eating" OR DE "Eating Attitudes" |
| S10 | (barrier* or obstacle* or difficult* or reason* or factor*) |
| S11 | (enabler* or facilitator* or determinan* or motivator*) |
| S12 | S1 OR S2 OR S3 OR S4 OR S5 OR S6 OR S7 OR S8 OR S9 OR S10 OR S11 |
| S13 | (DE "Nutrition" OR DE "Diets" OR DE "Food" OR DE "Weight Control") AND mediterran* |
| S14 | (diet* and mediterran*) |
| S15 | TI (MD) or AB (MD) |
| S16 | (TEAM-MED or MEDAS or med-diet) |
| S17 | (mediterranean N5 (lifestyle or life-style)) |
| S18 | (S13 OR S14 OR S15 OR S16 OR S17) |
| S19 | (T2DM or niddm or diabet*) |
| S20 | hyperglycaemi* or hyperglycemi* |
| S21 | DE "Type 2 Diabetes" OR DE "Blood Sugar" |
| S22 | DE "Hyperglycemia" |
| S23 | (S19 OR S20 OR S21 OR S22) |
| S24 | (S12 AND S18 AND S23) |

1. **Database: Web Of Science**

**dropout or drop-out or refusal or complian* or non-complian* or adheren* or non-adheren* or refusal or accept* or satisfaction or preference or determinan* or motivator or enabler** (All Fields) and **mediterran* diet or med-diet** (All Fields) and **diabetes or hyperglycemi* or hyperglycaemi* or NIDDM or T2DM** (All Fields)

1. Database: PubMed

| 11 | #4 AND #9 AND #10 |
| --- | --- |
| 10 | T2DM [tw] OR niddm [tw] OR diabet* [tw] OR "Diabetes Mellitus"[Mesh] OR "Diabetes Mellitus, Type 2"[Mesh] OR |
| 9 | #5 OR #6 OR #7 OR #8 |
| 8 | "diet"[MeSH Terms] AND "mediterran*"[Text Word] |
| 7 | "diet, mediterranean"[MeSH Terms] |
| 6 | "diet*"[Text Word] AND "mediterran*"[Text Word] |
| 5 | "med-diet"[Text Word] OR "MD"[Title/Abstract] OR TEAM-MED[tw] OR MEDAS [tw] OR Mediterranean lifestyle [tw] |
| 4 | #1 OR #2 OR #3 |
| 3 | "counseling"[MeSH Terms] OR "behavior therapy"[MeSH Terms] OR "patient education as topic"[MeSH Terms] OR |
| 2 | "barrier*"[Text Word] OR "obstacle*"[Text Word] OR "difficult*"[Text Word] OR "enabler*"[Text Word] OR "facilitator*"[ |
| 1 | "guideline adherence"[MeSH Terms |
