# Supplementary material for: Determinants of adherence to the Mediterranean diet among individuals with type 2 diabetes mellitus living in Mediterranean countries: a systematic review
Source: Front Nutr. 2025 Feb 3;12:1523995. doi: 10.3389/fnut.2025.1523995 (PMC11830624; doi:10.3389/fnut.2025.1523995)
Supplement: Supplementary file 4 [file Table_4.DOCX]

| **Table 1: Quality assessment of the included studies based on the Newcastle Ottawa Scale (NOS)** | | | | | | |
| --- | --- | --- | --- | --- | --- | --- |
| **Studies (Cross-sectional and observational)** | **Selection (Max. 5 stars)** | **Comparability: (Maximum 2 stars)** | **Main outcome (adherence to Med Diet) (Maximum 3 stars)** | **Final score/10** | **Risk of Bias** |  |
| (El Achhab et al., 2022)^29^ | 2 | 1 | 2 | 5 | High |  |
| (Grahovac et al., 2021)^30^ | 5 | 2 | 2 | 9 | Low |  |
| (Giugliano et al., 2010)^35^ | 3 | 2 | 2 | 7 | Medium |  |
| (Vidal-Peracho et al., 2017)^36^ | 4 | 2 | 2 | 8 | Low |  |
| (Roldan et al., 2019)^4^ | 2 | 2 | 2 | 6 | Medium |  |
| (Kudret et al.,2023)^24^ | 2 | 2 | 2 | 6 | Medium |  |
| (Yilmaz and Yangilar.,2022)^37^ | 3 | 1 | 2 | 6 | Medium |  |
| (Sanchez-Hernandez et al., 2020)^38^ | 4 | 1 | 2 | 7 | Medium |  |
| (Bucan Nenadic et al., 2022)^39^ | 4 | 2 | 2 | 8 | Low |  |
| (Ortega et al., 2013)^40^ | 5 | 2 | 2 | 9 | Low |  |
| (Badrasawi et al., 2021)^41^ | 5 | 1 | 2 | 8 | Low |  |
| (Alcubierre et al., 2020)^42^ | 4 | 2 | 2 | 8 | Low |  |
| (Muñoz-Pareja et al., 2012)^43^ | 3 | 1 | 2 | 6 | Medium |  |
| (Martinez-Gonzalez et al., 2012)^22^ | 5 | 2 | 2 | 9 | Low |  |
| (Downer et al., 2016)^44^ | 4 | 2 | 2 | 8 | Low |  |
| **Studies (Cohort)** | **Selection** | **Comparability: (Maximum 2 stars)** | **Outcome** | **Final score/9** | **Risk of bias** |  |
| (Bonaccio et al., 2016)^45^ | 3 | 2 | 3 (4 years) | 8 | Low |  |
| (Petroni et al., 2019)^31^ | 2 | 2 | 3 (2years) | 7 | Medium |  |
