# Supplementary material for: Determinants of adherence to the Mediterranean diet among individuals with type 2 diabetes mellitus living in Mediterranean countries: a systematic review
Source: Front Nutr. 2025 Feb 3;12:1523995. doi: 10.3389/fnut.2025.1523995 (PMC11830624; doi:10.3389/fnut.2025.1523995)
Supplement: Supplementary file 5 [file Table_5.DOCX]

| **Table 2: Categories of determinants affecting MD adherence in T2DM** | | |
| --- | --- | --- |
| **Demographic and personal Determinants** | **General Health and behavioral Determinants** | **Socioeconomic and physical determinants** |
| Gender | History of associated diseases | Habitation |
| Age | Physical activity |  |
| Education | Body Mass Index (BMI) |  |
| Marital status | Waist circumference (WC) |  |
| Therapeutic education | Duration of Diabetes |  |
|  | Smoking |  |
|  | Alcohol |  |
|  | Chronotype |  |
|  | Total energy intake (TEN) |  |
