# Supplementary material for: Determinants of adherence to the Mediterranean diet among individuals with type 2 diabetes mellitus living in Mediterranean countries: a systematic review
Source: Front Nutr. 2025 Feb 3;12:1523995. doi: 10.3389/fnut.2025.1523995 (PMC11830624; doi:10.3389/fnut.2025.1523995)
Supplement: Supplementary file 6 [file Table_6.DOCX]

| **Table 3: Mean adherence scores with the MD scoring tool and its respective classification in the included studies** | | | |
| --- | --- | --- | --- |
| **Reference - Country** | **MD soring tool** | **Range of MD adherence score for classification in categories** | **Mean adherence (Mean score ±SD)** |
| (Martinez-Gonzalez et al., 2012)^22^- Spain | MEDAS | Low (score ≤ 5). Moderate (score 6-9). High (score ≥10) | 8.6 ± 2 for the general population, 8.5 ± 2 for those with T2DM, 8.5 ± 2 for females, and 8.7 ± 2 for males. |
| (El Achhab et al., 2022)^29^- Morocco | MEDAS | Low (score 0-7). High (score 8-14) | 7.8 ± 1.2. 59% of the sample had a score of ≥ 8 points. |
| (Vidal-Peracho et al., 2017)^36^- Spain | MEDAS | Low (score ≤ 5). Moderate (score 6-9). High (score ≥10) | 8.7 ± 1.82 for the whole population. 8.5 ± 1.81 for those with T2DM. |
| (Roldan et al., 2019)^4^- Spain | MEDAS | Low ≤ 8. Good ≥ 9 | 6.2 ± 0.62 pre-education. 6.8 ± 0.62 post-education. A score of 6.5 for women and 5.8 for men |
| (Kudret et al.,2023)^24^- Turkey | MEDAS | Low (score ≤ 5). Moderate (score 6-9). High (score ≥10) | No mean adherence score. 39.0% of patients had low compliance. 56.5% had moderate compliance, and 4.5% had high compliance. 30% of men had low compliance versus 46.4% of women, 65.6% of men had moderate compliance versus 56.5% of women, and 4.4% of men had high adherence versus 4.5% of women. |
| (Yilmaz and Yangilar.,2022)^37^- Turkey | MEDAS | Low (score ≤ 5). Moderate (score 6-9). High (score ≥10) | The median PREDIMED score was 6. 46.3% had low adherence, 51.7% had medium and 2.0% had high. |
| (Sanchez-Hernandez et al., 2020)^38^- Spain | MEDAS | Low-to-moderate (score <9 points) or moderate-to-high (Score ≥9 points). | The mean score of MEDAS was not reported. 49.4% have poor/moderate adherence. 49.3% of men versus 49.4% of women had poor/moderate adherence. |
| (Downer et al., 2016)^44^- Spain | MEDAS | High (score ≥11) and low (score <11) | At 1-year follow-up, the mean score for low adherence was 8.2±1.8 and the mean score for high adherence was 9.3 ±1.8. 82% of patients had low compliance at 2-year follow up and 81% had low adherence at 3-year follow-up. At the 4-year follow-up, the mean score for low adherence was 8.5±2 and the mean score for high adherence was 9.3 ±9. |
| (Badrasawi et al., 2021)^41^- Palestine | MEDAS | Low adherence (score ≤5); moderate adherence (score between 6 and 9); and high adherence (score ≥10). | 53.8 % had low/moderate categories of adherence and 46.2% had high adherence categories. The average score of the high adherence group was 10.8±0.9, while that of the low/moderate group was 7.4±1.4. 59% of the male patients reported high adherence compared to just 38.8% of the females. |
| (Muñoz-Pareja et al., 2012)^43^- Spain | MEDAS | score from 0-14 with moderate accordance given to a score of ≥7. | MEDAS mean score was 6.8 (95% CI 6.65–7.02), and 57.4% (95% CI 52.6–62.3%) had accordance with MEDAS. Excluding alcohol, the MEDAS score increased to 74.9% (95% CI 70.3–79.3%). |
| (Giugliano et al., 2010)^35^- Italy | 9-point scale adapted by Trichopoulo et al. | 3 subgroups: low (score of 0–3), middle (4-5), and high (6–9). | No mean adherence to the Med Diet was reported. 28% had low adherence, 48% had moderate adherence and 24% had high adherence. |
| (Bonaccio et al., 2016)^45^- Italy | 9-point scale adapted by Trichopoulo et al. | 3 subgroups: low (score of 0–3), middle (4-5), and high (6–9). | Overall mean adherence was not mentioned. 30% of the population had poor adherence, 44% had moderate adherence and 26% had high adherence. The percentage of men was 66.1% of whom 27% had low adherence, 45.3% moderate, and 27.8% high. |
| (Ortega et al., 2013)^40^- Spain | Panagiotakos score (ATTICA study) | A score of 5 to 0 for each item. Low adherence (score<23); Medium (between 23-26); high (>26). | The mean (±SD) med score was 24 ±5. Of the general population, 36.5% had a low adherence (57% were women); 31% had a medium adherence (60% were women) and 32.4% had a high adherence (54% female) |
| (Alcubierre et al., 2020)^42^- Spain | Alternate Mediterranean Diet (aMED) score. | aMED: includes nine key components of the traditional MedDiet: The score ranges from 0 (minimal adherence) to 9 (maximal adherence).3 tertiles: T1 (score of 0-3); T2 (score of 3-6) and T3 (score of 6-9) | The mean aMED score was: 4.3 (1.5). Of 238 type 2 Diabetic patients: 30.3% were in T1; 47.9% in T2 and 21.8% in T3. |
| (Grahovac et al., 2021)^30^- Croatia | Med Diet service score (MDSS) | A score from 0 to 24. A score of over 13.5 points is considered good adherence to Med Diet principles. | The median score of adherence is 8.0 (6.0-10.0). (9.9%) patients fulfilled the criteria for adherence to MD. |
| (Bucan Nenadic et al., 2022)^39^- Croatia | Mediterranean Diet Serving Score (MDSS). | Score from 0 to 24. Optimal adherence with a score >14pts | The mean MDSS score was 8.3±3.45. 8.9% scored 14 or more points on total MDSS. 8.3% of patients with T2DM versus 9.1% of patients with CKD and T2DM had a score of 14 or more on MDSS. |
| (Petroni et al., 2019)^31^- Italy | Med diet score (MDS- 11 items) | Optimal, good, fair, scarce, and low at the respective cut-offs: ≥45; 44-34; 33-23; 22-12; ≤ 11 | MDS score was 29.6 ± 5.7 in the total population. 29.6 ± 5.4 in women and 29.7 ± 6 in men. |
